# Successful Surgical Treatment of Hepatocellular Carcinoma Invading Into Biliary Tree

**DOI:** 10.1155/1991/80824

**Published:** 1991

**Authors:** Kazuo Tanoue, Takashi Kanematsu, Takashi Matsumata, Ken Shirabe, Keizo Sugimachi, Chikao Yasunaga

**Affiliations:** 1 The Second Department of Surgery Faculty of Medicine Kyushu University 3-1-1 Maidashi, Higashi-ku Fukuoka 812 Japan; 2 The First Department of Pathology Faculty of Medicine Kyushu University Fukuoka Japan

## Abstract

A 41-year-old woman was admitted to hospital with obstructive jaundice. Computed tomography showed a large mass in the right hepatic lobe and marked dilatation of the biliary tree in the left lateral segment of the liver. Angiography showed evidence of neovascularity. Percutaneous transhepatic cholangiography revealed complete obstruction of the common bile duct just below the bifurcation. The serum level of alpha-fetoprotein on admission was 1,080,000 ng/ml. These findings suggested to us a primary hepatocellular carcinoma invading the intrahepatic bile duct. Extended right lobectomy and hepaticojejunostomy for bile drainage was carried out. The patient is doing well 3 years after surgery.

Hepatocellular carcinoma (HCC) invading to the portal vein is not so rare, but invasion into the bile duct is much less common. In 1947, Mallory^1^ described a single case of HCC invading the gallbladder and obstructing extrahepatic bile ducts. In 1975, Lin^2^ termed this HCC “Icteric type hepatoma”. The incidence of such HCC in Japan was reported to be 1.9-9%^2,3^.

Obstructive jaundice is a clinical manifestation of the terminal stage in HCC. We describe here our treatment of a woman with HCC invading the common bile duct. Right extended lobectomy and reconstruction of hepaticojejunostomy were effective.


*HPB Surgery*, 1991, Vol. 4, pp. 237–244
Reprints available directly from the publisher
Photocopying permitted by license only

© 1991 Harwood Academic Publishers GmbH
Printed in the United Kingdom

### CASE REPORT

### SUCCESSFUL SURGICAL TREATMENT OF HEPATOCELLULAR CARCINOMA INVADING INTO BILIARY TREE

KAZUO TANOUE, TAKASHI KANEMATSU, TAKASHI MATSUMATA, KEN SHIRABE and KEIZO SUGIMACHI
*The Second Department of Surgery, Faculty of Medicine, Kyushu University, Fukuoka, Japan*

CHIKAO YASUNAGA
*The First Department of Pathology, Faculty of Medicine, Kyushu University, Fukuoka, Japan*

*(Received 5 February 1991)*

A 41-year-old woman was admitted to hospital with obstructive jaundice. Computed tomography showed a large mass in the right hepatic lobe and marked dilatation of the biliary tree in the left lateral segment of the liver. Angiography showed evidence of neovascularity. Percutaneous transhepatic cholangiography revealed complete obstruction of the common bile duct just below the bifurcation. The serum level of alpha-fetoprotein on admission was 1,080,000 ng/ml. These findings suggested to us a primary hepatocellular carcinoma invading the intrahepatic bile duct. Extended right lobectomy and hepaticojejunostomy for bile drainage was carried out. The patient is doing well 3 years after surgery.

Hepatocellular carcinoma (HCC) invading to the portal vein is not so rare, but invasion into the bile duct is much less common. In 1947, Mallory[1] described a single case of HCC invading the gallbladder and obstructing extrahepatic bile ducts. In 1975, Lin[2] termed this HCC "Icteric type hepatoma". The incidence of such HCC in Japan was reported to be 1.9–9%[2,3].

Obstructive jaundice is a clinical manifestation of the terminal stage in HCC. We describe here our treatment of a woman with HCC invading the common bile duct. Right extended lobectomy and reconstruction of hepaticojejunostomy were effective.

KEY WORDS: Obstructive jaundice, hepatocellular carcinoma, hepatoma-induced biliary obstruction

A 41-year-old-Japanese woman noticed jaundice of the conjunctiva in October, 1987. She was seen by a local physician and obstructive jaundice due to a large tumor of the right lobe of the liver was diagnosed, using ultrasonography (US) and computed tomography (CT). She was referred to our clinic on November 18, 1987. The patient had no previous history of liver disease and had never received a blood transfusion.

Address correspondence to: Dr. Kazuo Tanoue, The Second Department of Surgery, Faculty of Medicine, Kyushu University, 3-1-1 Maidashi, Higashi-ku, Fukuoka 812, Japan.

On admission, physical examination revealed marked icterus. The abdomen was soft and flat. The liver, spleen and tumor were not palpable.

Laboratory chemistry showed a hemoglobin of 13.2 g/dl and hematocrit of 45%. The white blood cell count was 5670/mm$^3$. Prothrombin time was 10.4 seconds with a control of 11.4 seconds. Serum total bilirubin was 10.0 mg/dl, and direct bilirubin was 7.4 mg/dl. Serum glutamic oxaloacetic transaminase was 350 U/L, and serum glutamic pyrvic transaminase 564 U/L. The serum level of alpha-fetoprotein (AFP) was markedly elevated at 1,080,000 ng/ml. Hepatitis B surface antigen in the serum was positive.

Percutaneous transhepatic cholangiography (PTC) demonstrated an iregular defect from the main bifurcation of the hepatic bile duct to common bile duct (Figure 1). The left hepatic bile duct was intact and frequent cytologic examinations of the bile from the PTCD tube were all negative.

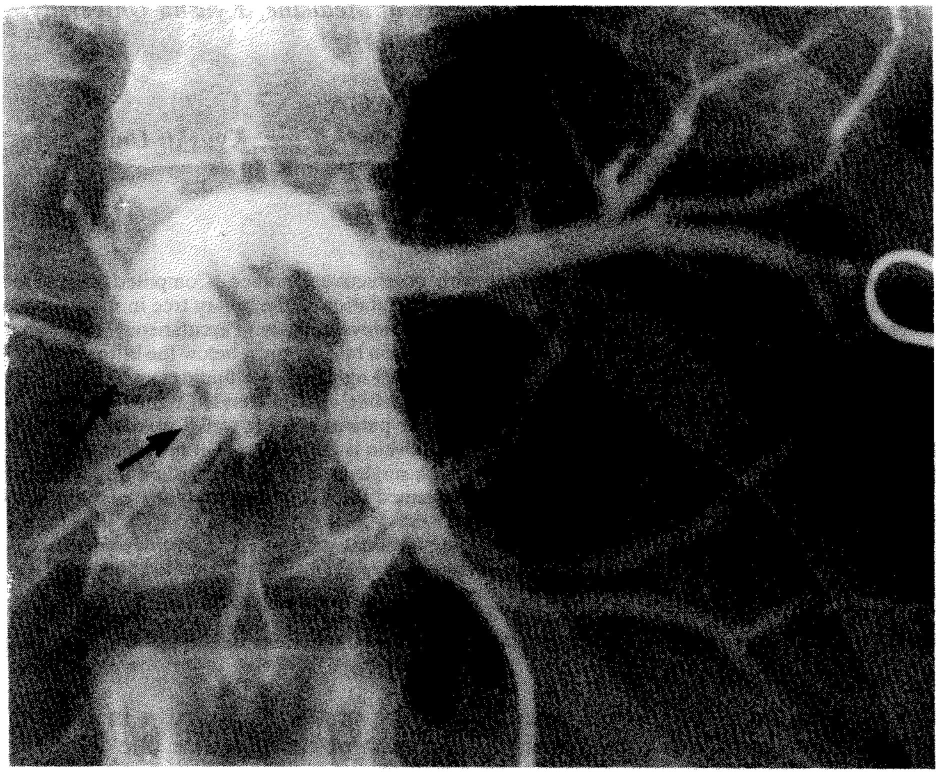

**Figure 1** Percutaneous transhepatic cholangiography. Note irregular defect from the main bifurcation of hepatic bile duct to common bile duct (arrows). Right hepatic duct cannot be seen due to obstruction by the tumor.

CT and US revealed a 12 × 10 cm tumor which occupied the entire right lobe of the liver was dilated the intrahepatic bile duct in the left lateral segment (Figure 2).

Celiac angiography revealed a large hypervascular mass in the right lobe of the liver (Figure 3). A mixture of 10 ml of lipiodol and 50 mg of adriamycin (ADM)

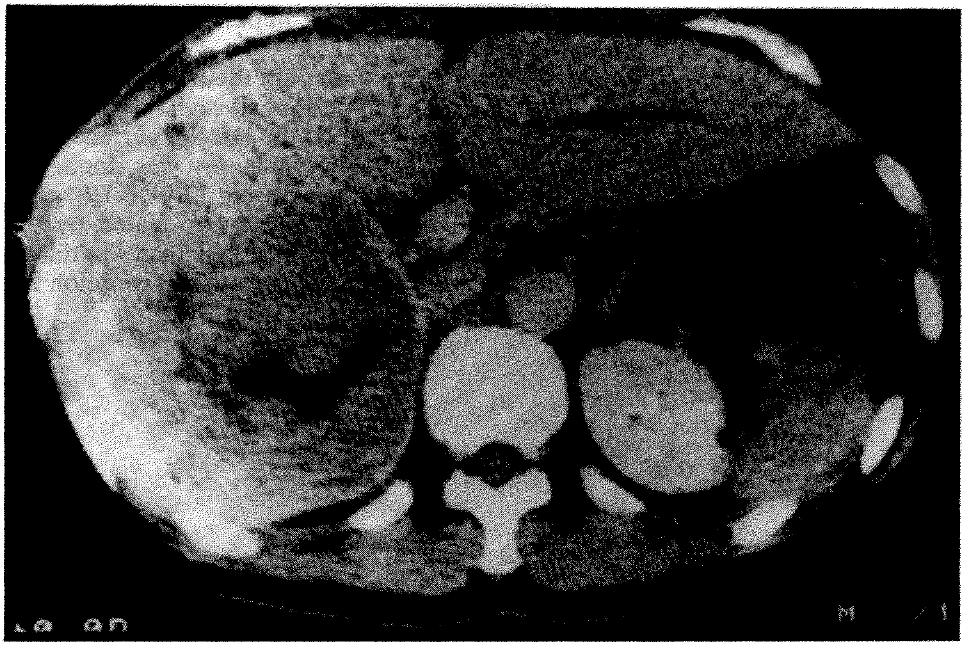

**Figure 2** Preoperative computed tomography. The huge tumor occupied the entire right lobe of the liver, and the left hepatic bile duct was dilated.

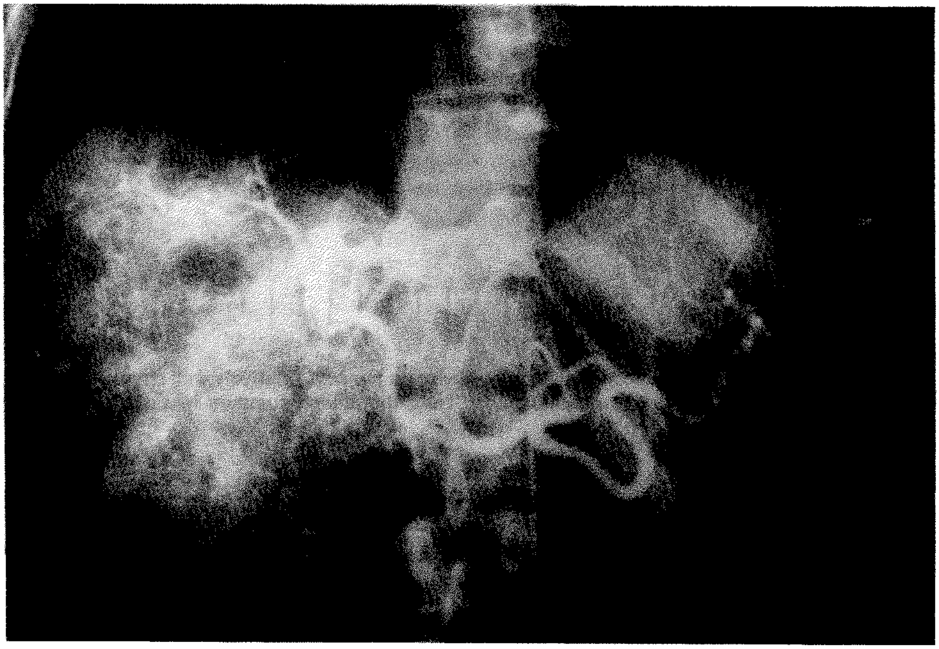

**Figure 3** Preoperative celiac angiography. Note the extensive hypervascularity in the right lobe of the liver.

was injected through a catheter inserted into the right hepatic artery, so called "lipiodolization"[4], as a preoperative chemotherapy.

On December 14, 1987, she underwent surgery, based on the diagnosis of HCC invading the bile duct. There were no ascites in the peritoneal cavity and the liver appeared grossly normal. A fist size tumor was found on the inferior surface of the right lobe. Extended right lobectomy plus hepaticojejunostomy was done. The resected specimen was a relatively demarcated mass measuring 12.0 × 7.6 × 7.8 cm (Figure 4). On cut section, the tumor was white and encapsulated by thin fibrous tissue. Extracapsular invasion was grossly evident. The bile duct was dilated and impacted by tumor tissue from the right hepatic bile duct to the common bile duct.

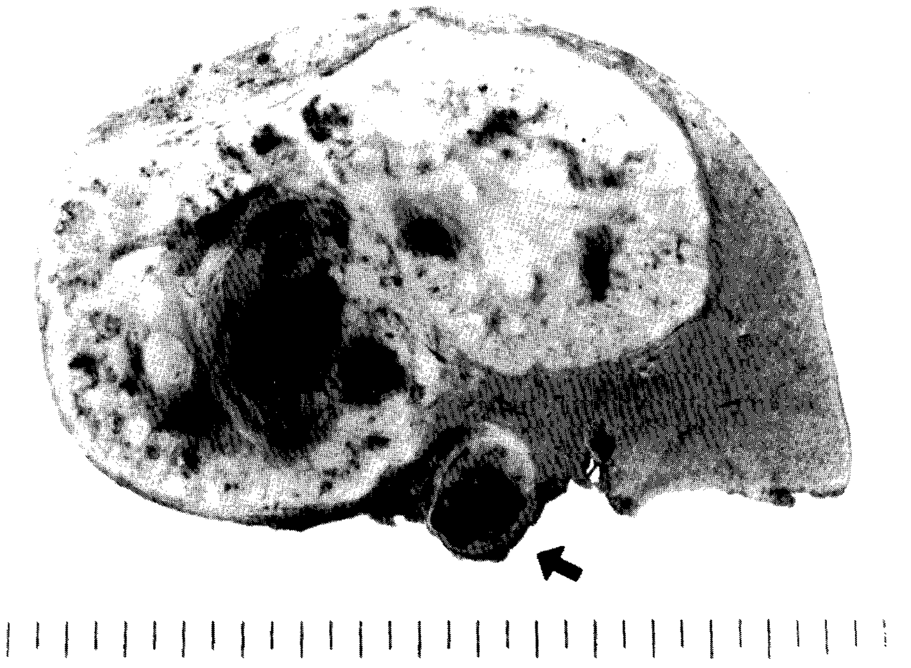

**Figure 4** The cut surface of the resected specimen. The main tumor is relatively well circumscribed by a thin fibrous capsule and shows central hemorrhage. Note the dilated common bile duct impacted by tumor tissue (arrow).

Microscopically, the tumor cells showed a thick-trabecular and partly pseudoglandular growth of HCC with marked pleomorphism (Figure 5a). In the common bile duct, the tumor had directly invaded the bile duct wall, although the biliary epithelium remained in parts (Figure 5b). Invasion into the portal vein was also evident. The non-cancerous tissue was present in the mildly enlaged portal tracts by proliferated bile ductules, a chronic inflammatory infiltrate and some bile-laden macrophages. There was neither hepatic change nor evidence of alcohol-related damage.

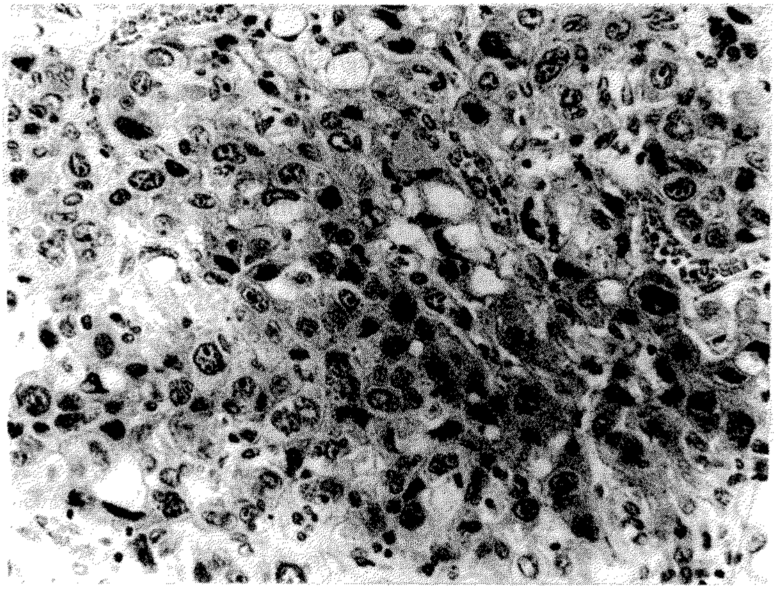

**Figure 5a** Photomicrograph of the tumor. Hepatocellular carcinoma showing marked pleomorphism (hematoxylin-eosin, ×230).

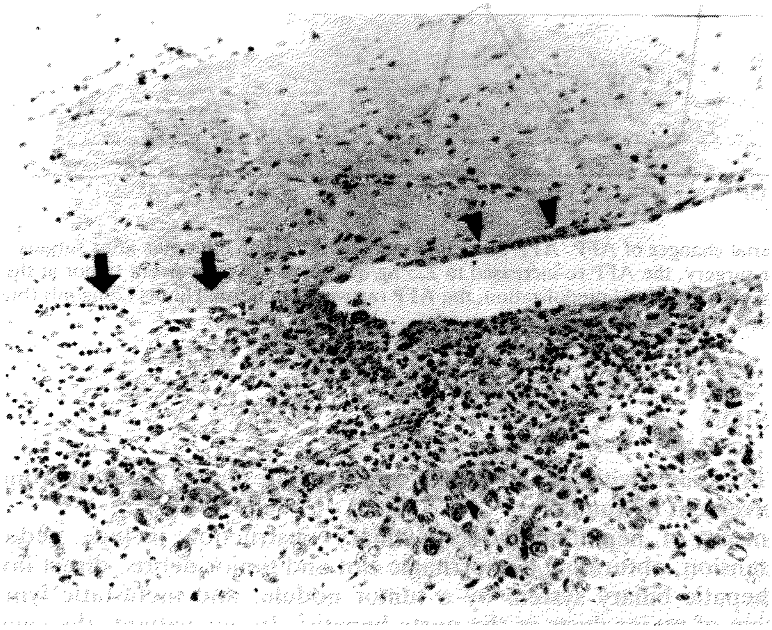

**Figure 5b** The wall of the common bile duct is invaded by tumor tissue (arrows). The bile epithelium is focally remaining (arrowheads).

On the 7th postoperative day, the total bilirubin decreased to within normal limits.

AFP gradually decreased from 1,080,000 ng/ml to 20 ng/ml within 3 months after the surgery, and she was completely rehabilited. About 8 months after the surgery, celiac angiography was performed because of a re-increase of AFP (225 ng/ml). A recurrent 2cm tumor was noted at the stump of the remnant liver. Lipiodolization using adriamycin 30 mg was prescribed, and the AFP decreased to within normal limits (Figure 6). The patient is doing well at this writing, 3 years after the surgery.

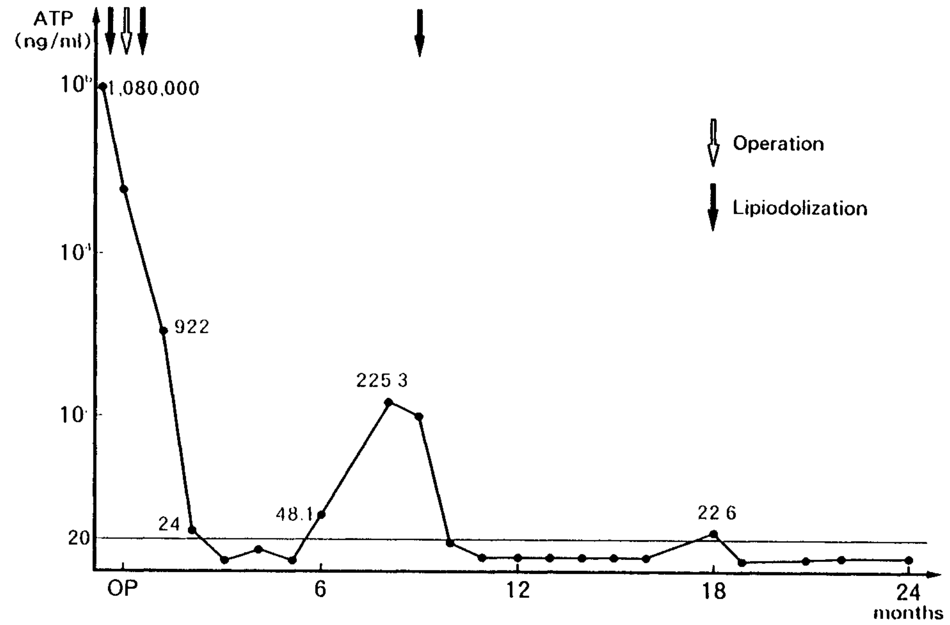

**Figure 6** Serial changes of AFP. AFP decreased to 20 ng/ml within 3 months after surgery. At the 8th month after surgery, the AFP re-increased to 225 ng/ml because of a recurrent tumor at the site of the anastomosis. After the 3rd lipiodolization, the AFP recovered in normal limits (20ng/ml) (black arrows: lipiodolization, open arrow: operation).

### DISCUSSION

Jaundice in patients with HCC is usually a late manifestation resulting from massive liver involvement by tumor.

Mechanisms of hepatoma-induced biliary obstruction include pedunculated tumor extension, obturating hemorrhagic clot and tumor debris, direct invasion of the intrahepatic biliary system by a tumor nodule, and metastatic lymph node compression of major ducts in the porta hepatis[5]. In our patient, the cause of the obstructive jaundice was direct invasion of HCC into the hepatic tree. In Japan, 58 cases of HCC complicated by extrahepatic obstructive jaundice had been reported up to the end of 1985[6].

The diagnosis of obstructive jaundice is facilitated by PTC and endoscopic retrograde cholangiopancreatography. The characteristic feature of bile duct involvement is a bulky intraluminal filling defect, most frequently located in the common hepatic duct. PTC in our patient revealed a defect in the right hepatic bile duct to the common hepatic duct. Frequent cytologic examinations of the bile from PTCD tube in the contralateral bile duct were negative.

Kojiro *et al.*[3] reported that HCC with bile duct invasion showed infiltrative growth into the surrounding tissue in all of 24 cases. This growth pattern seems to be responsible for the relatively poor prognosis of the HCCs with bile duct invasion, compared with those without such invasion. The tumor in our patient was well encapsulated but associated capsular invasion and permeation in to the portal veins was evident, even macroscopically.

The ideal treatment of these patients is complete extirpation of the tumor by hepatic resection. Unfortunately, the majority of tumors are unsuitable for resection because tumor is often in close proximity to the hepatic hilum[7]. Hepatic resection has only been reported occasionally[8,15]. In Japan, curative liver resection was performed in only 10 patients as of the end of 1985[6]. The surgical procedure for patients in whom curative liver resection was not possible has been choledochotomy with biliary drainage by T-tube or a bypass of the biliary tract such as choledochojejunostomy[6–19]. In our institution, we have performed liver resection for hepatoma in 235 patients in 10 years, however it was only in this patient that extended right lobectomy plus hepaticojejunostomy as a curative operation was successful for this type of HCC.

We wish to emphasize that hepatic resection even in patients with HCC invading the biliary tree can be successful, when the tumor does not occupy the opposite lobe or segment and there are no tumor cells in the bile obtained through the PTC catheter.

### *Acknowledgment*

We thank M. Ohara for critical comments.

### *References*

1. Mallory, T.B. (1947) Case records of the Massachusetts General Hospital. *N. Engl. J. Med.*, **237**, 673–676
2. Lin, T.Y. (1976) Tumors of the liver. In: Bockus, H.L., ed. *Gastroenterology*, pp. 522–533. Philadelphia: WB Saunders
3. Kojiro, M., Kawabata, K., Kawano, Y., Shirai, F., Takemoto, N. and Nakashima, T. (1982) Hepatocellular carcinoma presenting as intrabile duct tumor growth. *Cancer*, **49**, 2144–2147
4. Kanematsu, T., Furuta, T., Takenaka, T., Matsumata, T., Yoshida, Y., Nishizaki, T., Hasuo, K. and Sugimachi, K. (1989) A 5-year experience of lipiodolization: Selective regional chemotherapy for 200 patients with hepatocellular carcinoma. *Hepatology*, **10**, 98–102
5. vanSonnenberg, E., Ferrucci, J.T., Jr. (1979) Bile duct obstruction in hepatocellular carcinoma. *Radiology*, **130**, 7–13
6. Lee, K.C., Sakai, K., Kinoshita, H., Hirohashi, K., Tsuji, Y. and Kubo, S. (1988) Resection of Hepatocellular Carcinoma with Obstructive Jaundice Caused by Compression of the Common Hepatic Duct. *J. Surg. Oncol.*, **39**, 201–205
7. Law, W.Y., Leung, J.W.C. and Li, A.K.C. (1990) Management of hepatocellular carcinoma presenting as obstructive jaundice. *Am. J. Surg.*, **160**, 280–282
8. Tsuzuki, T., Ogata, Y., Iida, I., Kasajima, M. and Takahashi, S. (1979) Hepatoma with obstructive jaundice due to the migration of a tumor mass in the biliary tract: Report of a successful resection. *Surgery*, **85**, 593–598

9. Johns, W.A. and Zimmerman, A. (1961) Biliary obstruction due to hemobilia caused by liver cell carcinoma. *Ann. Surg.*, **153**, 706–710
10. Dickenson, A.J. and Santuli, T.V. (1962) Obstruction of common bile duct by hepatoma. *Surgery*, **52**, 800–802
11. Gerson, C.D. and Schinella, R.A. (1969) Hepatoma presenting as extrahepatic biliary obstruction. *Am. J. Dig. Dis.*, **14**, 42–47
12. Ishikawa, I., Kobayashi, K., Odajima, S., Takada, A. and Takeuchi, J. (1973) Primary hepatic cancer with recurrent episodes of obstructive jaundice and distended gallbladder. *Am. J. Gastroenterol.*, **60**, 496–503
13. Kuroyanagi, Y., Sawada, M., Hidemura, R., Aoki, S. and Kato, H. (1977) Common bile duct obstruction by hepatoma. *Am. J. Surg.*, **133**, 233–235
14. Gary, W. and Futterman, S. (1977) Obstructive jaundice secondary to hepatoma. *Am. J. Gastroenterol.*, **153**, 80–83
15. Afroudakis, A., Bhuta, S.M., Ranganath, K.A. and Kaplowitz, N. (1978) Obstructive jaundice caused by hepatocellular carcinoma. Report of three cases. *Am. J. Dig. Dis.*, **23**, 609–617
16. Jurco, S. and Kim, H.S. (1980) Extra biliary obstruction by hepatocellular carcinoma. *Am. J. Gastroenterol.*, **74**, 176–178
17. Nonomura, A., Ohta, G., Kanai, M. and Kobayashi, K. (1983) Hepatocellular carcinoma presenting extrahepatic biliary obstruction. *Acta. Pathol. Jpn.*, **33**, 789–806
18. Joehl, R.J. and Abt, A.B. (1984) Obstructive jaundice caused by hepatocellular carcinoma. *J. Surg. Oncol.,*, **27**, 80–84
19. Sarma, D.P., Weilbaecher, T.G. and Deipairine, E.M. (1987) Hepatocellular carcinoma causing obstructive jaundice. *J. Surg. Oncol.*, **34**, 192–193

*(Accepted by S. Bengmark 5 February 1991)*

